# Supplementary material for: Modeling brain sex in the limbic system as phenotype for female-prevalent mental disorders
Source: Biol Sex Differ. 2024 May 15;15:42. doi: 10.1186/s13293-024-00615-1 (PMC11097569; doi:10.1186/s13293-024-00615-1)
Supplement: Supplementary file 1 — Additional File 1: Supplementary Figures S1-S7, Supplementary Table 1. [file 13293_2024_615_MOESM1_ESM.docx]

**Additional File 1**
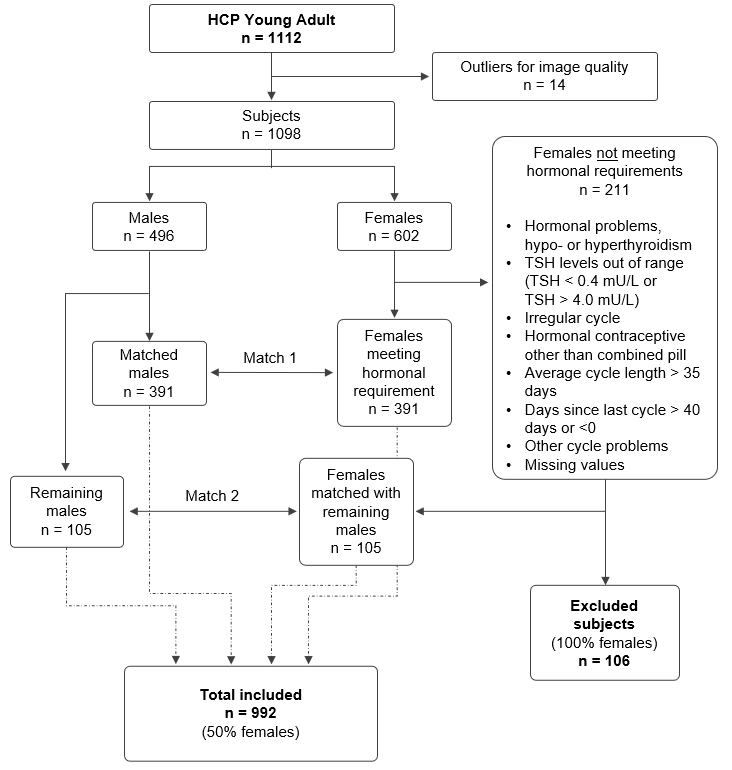


**Supplementary Figure S1. Double matching procedure for Human Connectome Project (HCP) sample.** After excluding outliers for image quality, female subjects were divided in two groups based on the hormonal information available. The group meeting the hormonal requirements was first matched according to age and image quality to a subgroup of males, to limit as much as possible the effects of hormonal fluctuation. Finally, to maximize the sample size, a second matching was applied between the remaining males and a subgroup of females not meeting the hormonal requirements. The final total sample size was N = 992 subjects, with an equal ratio between sexes. Abbreviations: TSH: Thyroid Stimulating Hormone


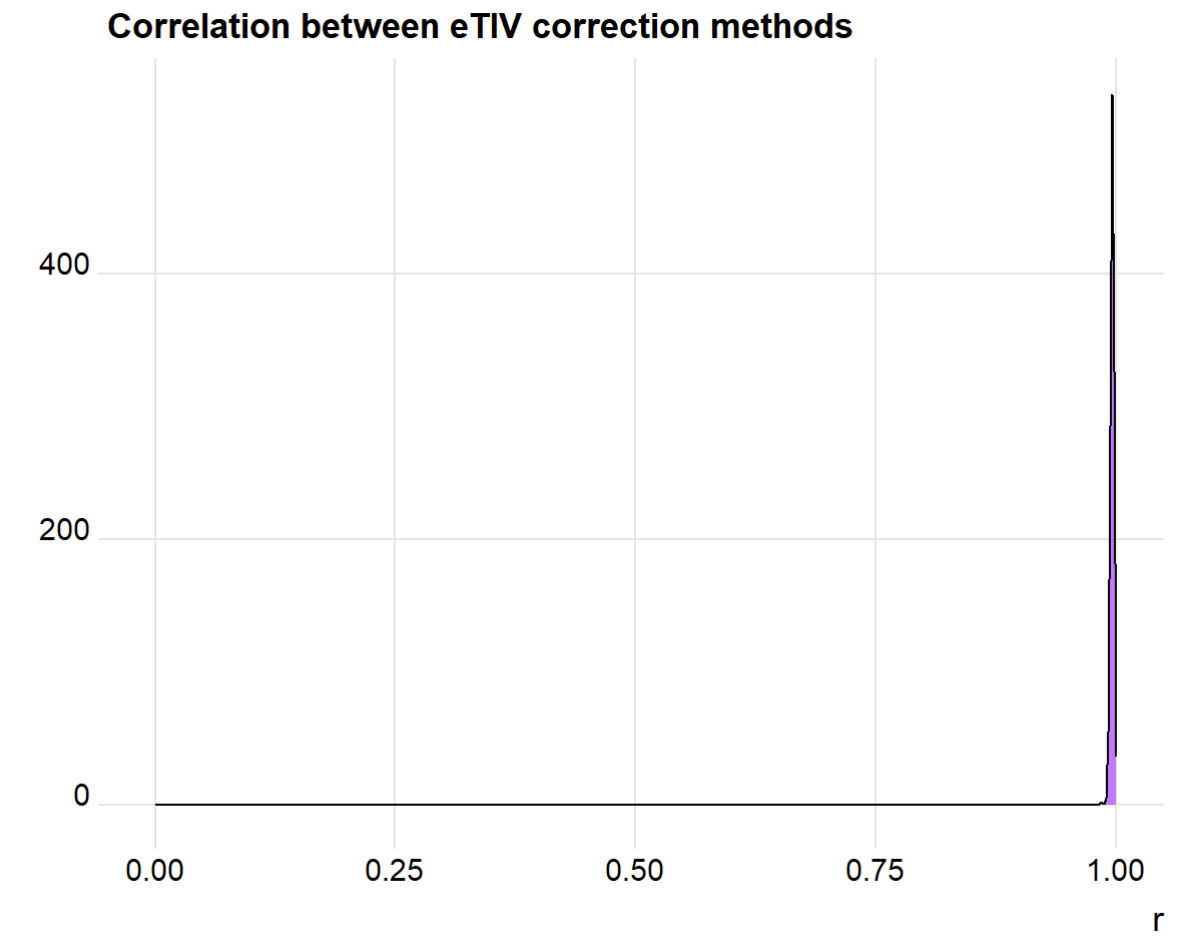


**Supplementary Figure S2. Different eTIV correction approaches converge.** Each brain feature was corrected for eTIV with two different methods, (1) using a residualisation approach, and (2) the power-corrected proportion method. For each feature, the results were correlated. The plot depicts the density across all correlation coefficients, indicating high convergence across the two tested approaches (minimum correlation r = 0.98).


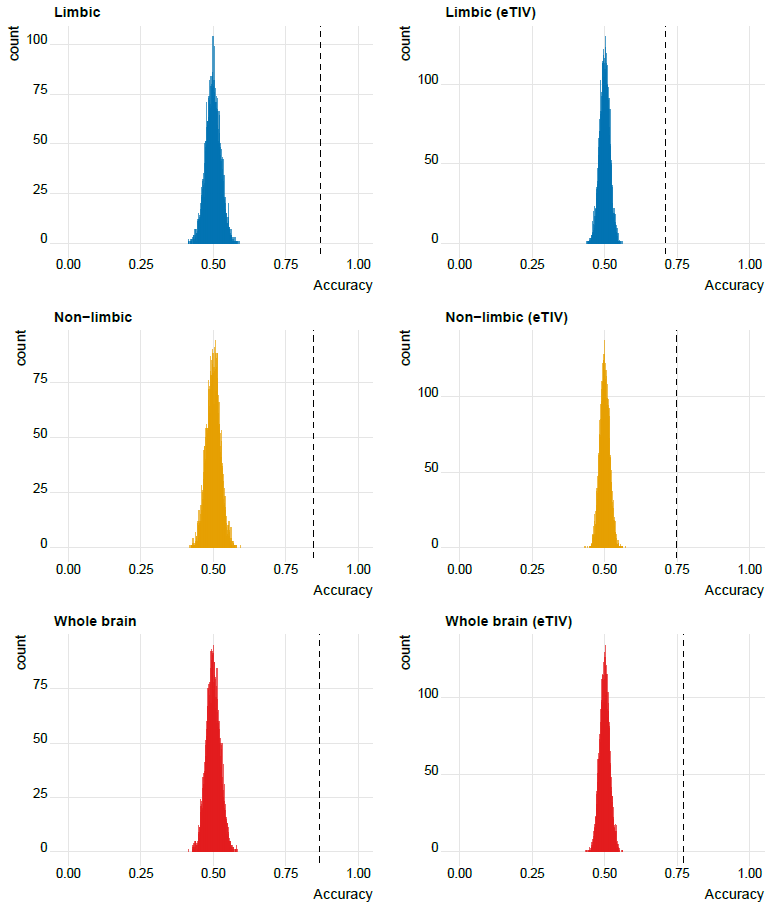


**Supplementary Figure S3. All model achieved significant accuracy.** No permutation-test based accuracy across 5000 permutations was higher than the accuracies achieved with the true model.


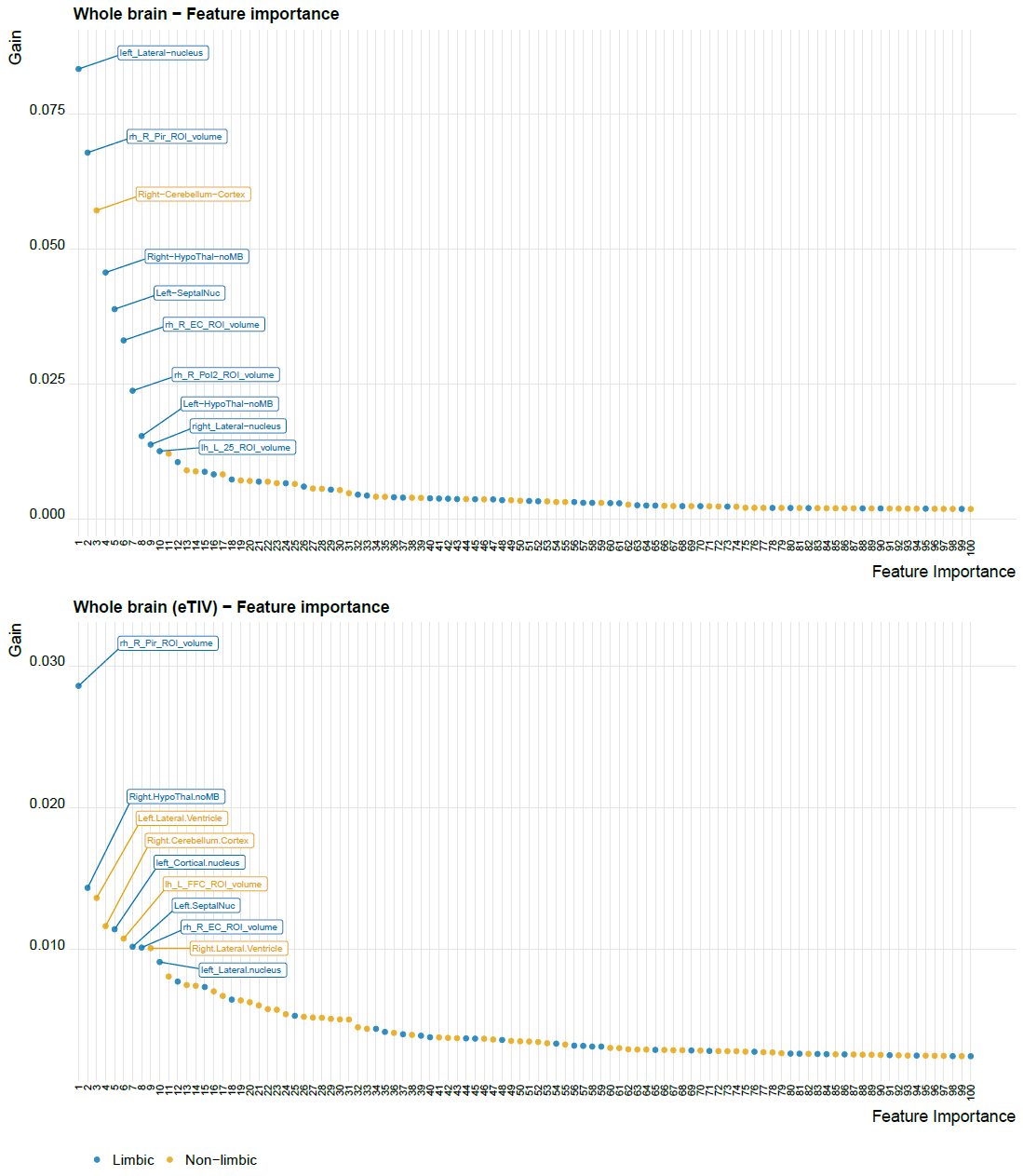


**Supplementary Figure S4. The majority of the 10 most important features of the whole brain models belong to the limbic system.** The feature importance of the first 100 features indicating the contribution of limbic and non-limbic structures.


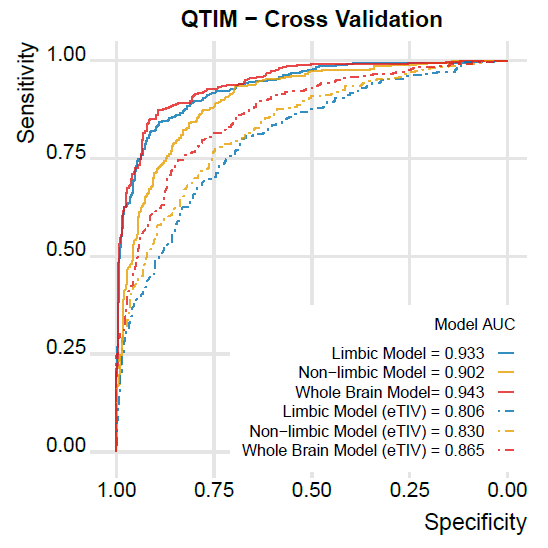


**Supplementary Figure S5. Training the model in QTIM achieved high performances for all models.** The accuracies and AUC obtained were comparable to those obtained in the HCP and when testing in QTIM.


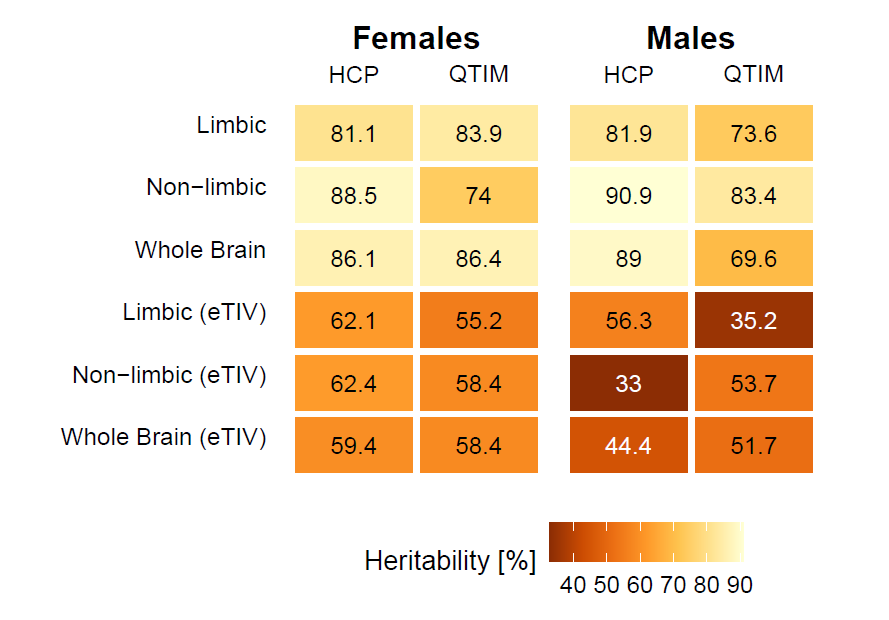


**Supplementary Figure S6. The class probabilities are heritable independently from sex.** Each cell shows the broad sense heritability in percent for females and males separately.


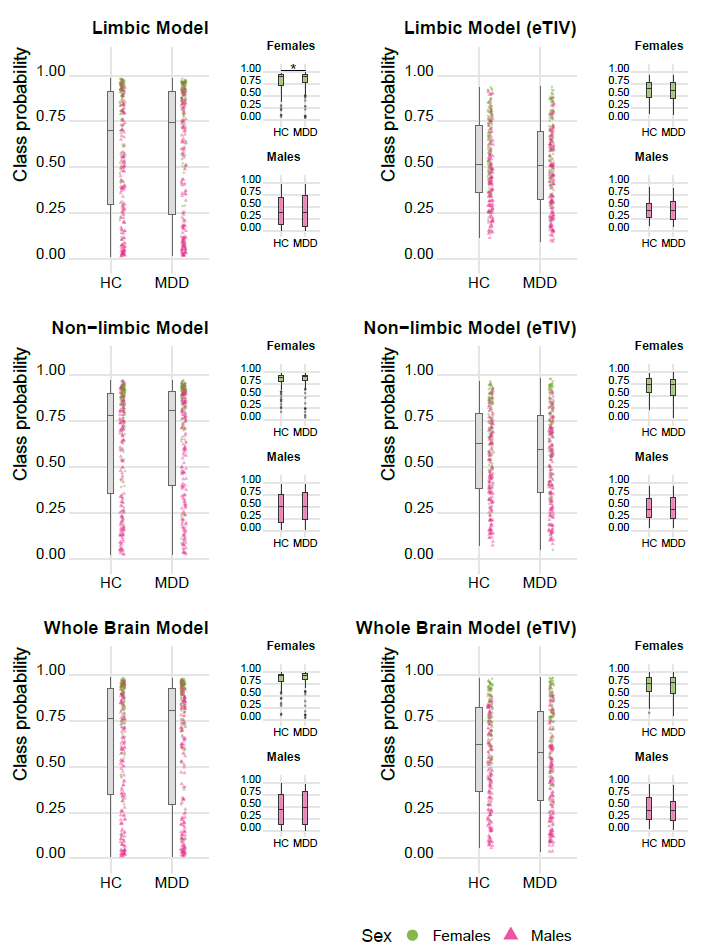


**Supplementary Figure S7. Limbic class probabilities in females are significantly higher in age-stratified analyses for the raw model.** When matching healthy controls (HC) and Major Depressive Disorders patients (MDD) according to age, MDD diagnosis is significantly associated with higher limbic class probabilities in females when considering the raw features. No significant effect was found for the other raw or eTIV-corrected model or in males. * p < 0.05

**Supplementary Table 1. List of limbic structures with the correspondent feature in Freesurfer**

| **Structure** | **FreeSurfer Feature** | **Segmentation** |
| --- | --- | --- |
| Anterior Cingulate Cortex | 33pr | Glasser atlas |
|  | p24pr |  |
|  | a24pr |  |
|  | p24 |  |
|  | a24 |  |
|  | p32pr |  |
|  | a32pr |  |
|  | d32 |  |
|  | p32 |  |
|  | s32 |  |
|  | 8BM |  |
|  | 9m |  |
|  | 10v |  |
|  | 10r |  |
|  | 25 |  |
| Orbitofrontal Cortex | OFC |  |
|  | pOFC |  |
| Insula | MI |  |
|  | AVI |  |
|  | AAIC |  |
|  | Ig |  |
|  | PI |  |
|  | PoI1 |  |
|  | PoI2 |  |
| Piriform Cortex | Pir |  |
| Entorhinal Cortex | EC |  |
| Parahippocampal Area | PHA1 |  |
|  | PHA2 |  |
|  | PHA3 |  |
| Posterior Cingulate Cortex | DVT |  |
|  | ProS |  |
|  | POS1 |  |
|  | POS2 |  |
|  | RSC |  |
|  | v23ab |  |
|  | d23ab |  |
|  | 31pv |  |
|  | 31pd |  |
|  | 31a |  |
|  | 23d |  |
|  | 23c |  |
|  | PCV |  |
| Hippocampus | Hippocampal_tail | Hippocampal Subfields |
|  | subiculum-body |  |
|  | CA1-body |  |
|  | subiculum-head |  |
|  | hippocampal-fissure |  |
|  | presubiculum-head |  |
|  | CA1-head |  |
|  | presubiculum-body |  |
|  | parasubiculum |  |
|  | molecular_layer_HP-head |  |
|  | molecular_layer_HP-body |  |
|  | GC-ML-DG-head |  |
|  | CA3-body |  |
|  | GC-ML-DG-body |  |
|  | CA4-head |  |
|  | CA4-body |  |
|  | fimbria |  |
|  | CA3-head |  |
|  | HATA |  |
| Amygdala | Lateral-nucleus | Nuclei of Amygdala |
|  | Basal-nucleus |  |
|  | Accessory-Basal-nucleus |  |
|  | Anterior-amygdaloid-area-AAA |  |
|  | Central-nucleus |  |
|  | Medial-nucleus |  |
|  | Cortical-nucleus |  |
|  | Corticoamygdaloid-transitio |  |
|  | Paralaminar-nucleus |  |
| Anterior and Dorsomedial Thalamic Nuclei | AV | Thalamic Nuclei |
|  | LD |  |
|  | MDl |  |
|  | MDm |  |
| Nucleus Accumbens | Nucleus-Accumbens | ScLimbic |
| Hypothalamus | HypoThal-noMB |  |
| Fornix | Fornix |  |
| Mammillary Body | MammillaryBody |  |
| Forebrain | Basal-Forebrain |  |
| Septal Nuclei | SeptalNuc |  |
